# Phase Structure, Bond Features, and Microwave Dielectric Characteristics of Ruddlesden–Popper Type Sr_2_TiO_4_ Ceramics

**DOI:** 10.3390/ma16145195

**Published:** 2023-07-24

**Authors:** Jun Yang, Jinbiao Pang, Xiaofang Luo, Laiyuan Ao, Qiang Xie, Xing Wang, Hongyu Yang, Xianzhong Tang

**Affiliations:** 1 School of Materials and Energy, University of Electronic Science and Technology of China, Chengdu 611731, China; 2 China Zhenhua Group Yunke Electronic Co., Ltd., Guiyang 550018, Chinaaly0764@163.com (L.A.); xoxq_z@163.com (Q.X.);; 3 China Zhenhua Group Xinyun Electronic Co., Ltd., Guiyang 550018, China; 4 Academy of Advanced Interdisciplinary Research, Xidian University, Xi’an 710071, China

**Keywords:** Sr_2_TiO_4_, microwave dielectric properties, P–V–L complex chemical bond theory

## Abstract

This work studied the phase constitution, bond characteristics, and microwave dielectric performances of Sr_2_TiO_4_ ceramics. Based on XRD and Rietveld refinement analysis, pure tetragonal Ruddlesden–Popper type Sr_2_TiO_4_ ceramic is synthesized at 1425~1525 °C. Meanwhile, the microstructure is dense and without porosity, indicating its high sinterability and densification. Great microwave dielectric performances can be obtained, namely an *ε_r_* value of 39.41, and a *Q × f* value of 93,120 GHz, when sintered at 1475 °C. Under ideal sintering conditions, the extrinsic factors are minimized and can be ignored. Thus, the intrinsic factors are considered crucial in determining microwave dielectric performances. Based on the P–V–L complex chemical bond theory calculation, the largest bond ionicity, and proportions to the bond susceptibility from Sr–O bonds suggest that Sr–O bonds mainly determine the dielectric polarizability. However, the Ti–O bonds show lattice energy about three times larger than Sr–O bonds, emphasizing that the structural stability of Sr_2_TiO_4_ ceramics is dominated by Ti–O bonds, and the Ti–O bonds are vital in determining the intrinsic dielectric loss. The thermal expansion coefficient value of the Sr_2_TiO_4_ structure is also mainly decided by Ti–O bonds.

## 1. Introduction

With the development of 5G technology, microwave components, including microwave circuits, dielectric antennas, dielectric resonators, and dielectric filters, are widely used for their advantages in terms of small size, lightweight nature, and large quality factor (*Q*) value [1,2]. The microwave components fabricated by microwave dielectric ceramic exhibit great potential and market application value. Theoretically, an ideal ceramic candidate should have an adjustable dielectric constant (*ε_r_*), an ideal dielectric loss (tan*δ*, tan*δ* = 1*/Q*), a high *Q × f* (*Q × f* refers to the *Q* value under a certain resonant frequency *f*) value, and a tunable temperature coefficient of resonance frequency (*τ_f_*) value [3]. Hence, it is urgent to search for material candidates with outstanding microwave dielectric performance [4].

Ceramic systems with medium *ε_r_* (20~70) values can reduce the device size and decrease the dissipation of microwave energy; as such, they have been attracting the attention of many scholars over the decades. Table 1 lists some popular ceramic materials that have been broadly investigated [5,6,7,8,9,10,11].

From Table 1, the niobate-based material systems have a large, coordinated range of dielectric constants and excellent *Q × f* value. Recently, a tetragonal Ruddlesden–Popper structure containing Sr_2_LaAlTiO_7_ (*ε_r_* = 26.5, *Q × f* = 110,850 GHz, *τ_f_* = 2.95 ppm/°C) [12], (Sr_1−*x*_Ca*_x_*)SmAlO_4_ (*ε_r_* = 18.7, *Q × f* = 125,000 GHz, *τ_f_* = −9 ppm/°C) [13], and Sr_n+1_Ti_n_O_3n+1_ (an *ε_r_* value of 42, a *Q × f* value~145,200 GHz, and a *τ_f_* value ca. of 130 ppm/°C for *n* = 1) [10] has also been reported with great microwave dielectric performances.

It is widely acknowledged that the microwave dielectric performances of a ceramic system are primarily controlled by the phase structure when the extrinsic dielectric loss is properly regulated [14]. Even though the Sr_2_TiO_4_ ceramic shows an ultra-high *Q × f* value, the present literature reports have not conducted in-depth research on the structure–performance correlation. Thus, its dielectric characteristics concerning crystal structure are still unclear. Therefore, we wonder which part of the crystal structure influences the dielectric performances at the microwave range. Is it possible to determine the influence from the Sr site and Ti site and give guidance for ionic modification? To test this, Sr_2_TiO_4_ microwave dielectric ceramic was synthesized in the present study, where its structural impacts on chemical bond traits and microwave dielectric performances were investigated comprehensively.

## 2. Materials and Methods

Raw fine powders of SrCO_3_ (Aladdin, 99.9%) and TiO_2_ (Aladdin, 99.9%) were proportionally blended in view of the chemical formula of Sr_2_TiO_4_ in a ball-mill tank with zirconia balls and deionized water for 5 h. After that, the mixture was dried and sieved using a 120-mesh screen. Then, it was calcined at 1200 °C for 4 h to produce the Sr_2_TiO_4_ phase. The calcined powder was secondary ball-milled for 5 h. After that, the combination was dried and added with a polyvinyl alcohol solution to form cylinders (diameter: 12 mm; thickness: 6 mm), and then the pellets were sintered at 1425~1525 °C for 4 h.

The phase structure is analyzed by a powder X-ray diffraction instrument (Philips X’Pert Pro MPD, PANalytical, Morvern, UK). The structural Rietveld refinement examination was followed by using the GSAS-EXPGUI package to obtain crystal structural parameters, including cell volume, axis length, chemical bond type, chemical bond length, and chemical bond angle [15,16]. Before performing the analysis, the refined order was strictly required following the background, peak shape parameter fitting, and thermal vibration factor. The microstructure of sintered specimen was detected using scanning electron microscopy (SEM, FEI Inspect F), where the grain size distribution was analyzed by Nanomeasurer 1.2 software. The bulk density was acquired by Archimedes’ method. The theoretical and relative density were calculated based on the following [17]:

(1) $$\rho_{theo}=\frac{n\times A}{N\times V_{cell}}$$

(2) $$\rho_{re}=\frac{\rho_{bulk}}{\rho_{theo}}$$

where *n*, *A*, *V_cell_*, and *N* are the number of molecules in a unit cell (the number of structural units equals 2 for the Sr_2_TiO_4_ structure), molecular weight (g/mol), the unit cell volume (cm^3^), and Avogadro number (mol^−1^), respectively. The microwave dielectric performances were examined using a Hakki–Coleman dielectric resonator method under the TE_011_ mode with a network analyzer (E5071C, Agilent Technologies Co., Ltd., Santa Clara, CA, USA). The *τ_f_* value was measured at 25 °C and 85 °C using the following formula:

(3) $$\tau_{f}=\frac{f_{85}-f_{25}}{60\cdot f_{25}}\cdot 10^{6}\left(\mathrm{ppm}/{}^{\circ}C\right)$$

where *f*_25_ and *f*_85_ are the resonant frequency of ceramics at 25 °C and 85 °C, respectively.

## 3. Results

### 3.1. Phase Structure Investigation of Sr_2_TiO_4_ Ceramic

**Figure 1:**
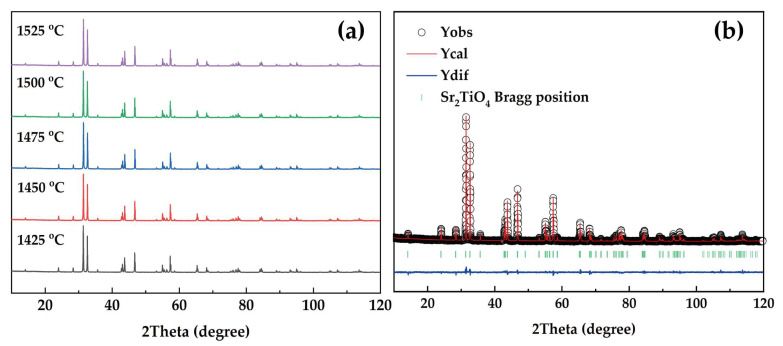
(**a**) XRD patterns of Sr_2_TiO_4_ ceramics sintered at 1425~1525 °C; (**b**) XRD profile of Sr_2_TiO_4_ ceramic sintered at 1475 °C after whole pattern fitting.

The X-ray diffraction (XRD) patterns of Sr_2_TiO_4_ ceramics sintered at 1425~1525 °C are shown in Figure 1a, and the XRD profile of Sr_2_TiO_4_ ceramic sintered at 1475 °C after the whole pattern fitting is presented in Figure 1b. All the observed diffraction peaks are assigned and indexed with JCPDS card No. 39-1471. No other peaks assigned to the second phase are noticed, suggesting a pure tetragonal Ruddlesden–Popper structure formation. The estimation criteria of *R_wp_* = 5.99, *R_p_* = 4.66, and *χ^2^* = 2.008 are suitable and in an acceptable range, indicating the Rietveld refinement analysis’s validity. The refined structural parameters are *a* = *b* = 3.8898 (4) Å, *c* = 12.6089 (3) Å, and a *V_cell_* of 190.779 (8) Å^3^ with an *I4/mmm*(139) space group.

**Figure 2:**
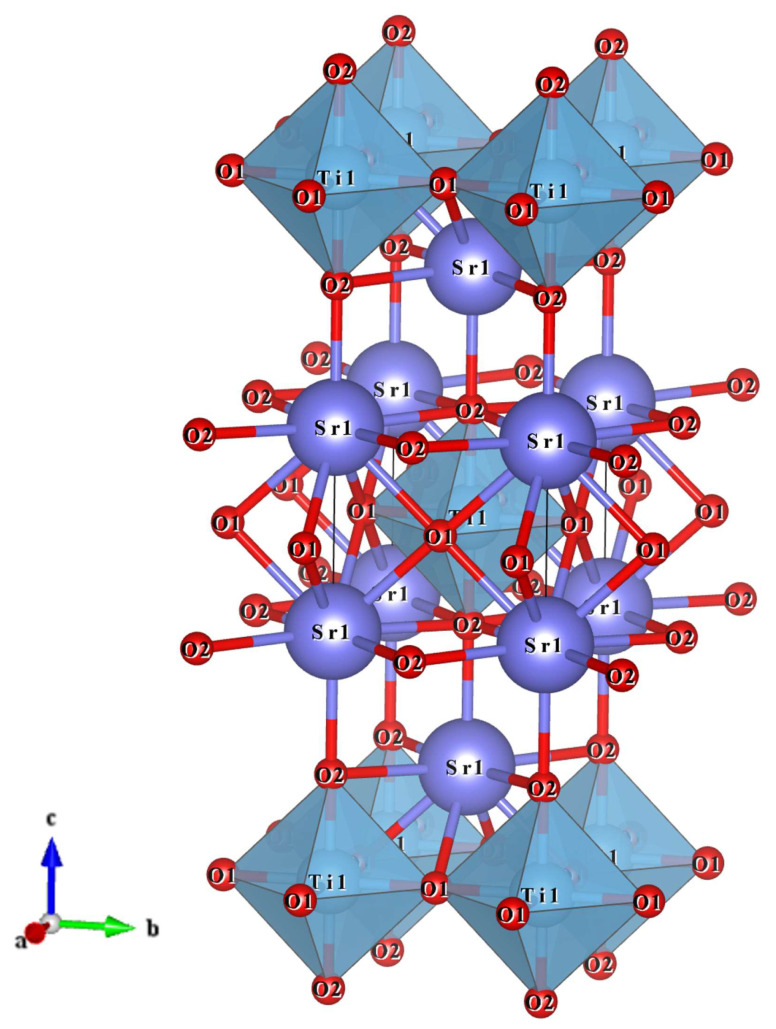
Schematic representation of Sr_2_TiO_4_ crystal structure.

The graphical representation of the Sr_2_TiO_4_ phase structure is shown in Figure 2. The Sr_2_TiO_4_ crystallizes in a tetragonal Ruddlesden–Popper structure with a space group of *I4/mmm*(139). It is treated as the arrangement of oxygen polyhedrons in an arrangement order of Ti–Sr–Sr–Ti–Sr–Sr–Ti along the *c*-axis direction. There are nine oxygen anions and six oxygen anions around the Sr^2+^ and Ti^4+^ cations, constructing a close-fitting bound [SrO_9_] and [TiO_6_] polyhedrons, where the [SrO_9_] polyhedrons are interconnected by sharing edges, and the [TiO_6_] octahedrons are interconnected by a shared vertex. The [SrO_9_] connects with [TiO_6_] through the edges. Particularly, three types of O anions occur in [SrO_9_]: Sr–O1 × 4 bonds, Sr–O2(1) × 4 bonds, and a Sr–O2(2) × 1 bond, while two types of O anions occur in [TiO_6_]: Ti–O1 × 4 bonds and Ti–O2 × 2 bonds. Exact atomic fractional coordinates and anion and cation spacing are listed in Table 2.

### 3.2. Microstructure Investigation of Sr_2_TiO_4_ Ceramic

**Figure 3:**
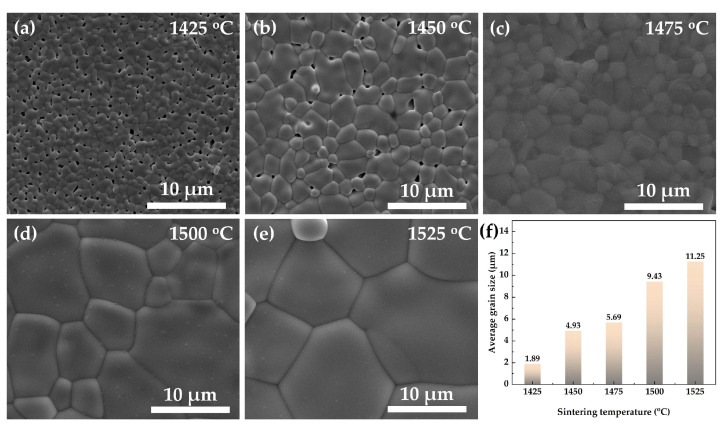
SEM images of Sr_2_TiO_4_ ceramics sintered at (**a**) 1425 °C, (**b**) 1450 °C, (**c**) 1475 °C, (**d**) 1500 °C, and (**e**) 1525 °C; (**f**) the variation in average grain size.

The SEM images of Sr_2_TiO_4_ ceramic sintered at 1425~1525 °C are presented in Figure 3. It is shown that when the sintering temperature is as low as 1425 °C, some observable micropores are accompanied by a small average grain size of about 1.89 μm, indicating that the Sr_2_TiO_4_ ceramic is not well-densified. As the temperature gradually increases from 1425 °C to 1475 °C, as shown in Figure 3a–c, the growth of grain is promoted and the amounts of micropores decline. Specifically, high densification can be achieved in Figure 3c, which suggests its high sinterability. It also shows a uniform distribution of grain size, where the largest, smallest, and mean grain sizes are about 5.69 μm, 1.19 μm, and 2.58 μm, respectively. The bulk density obtained via the Archimedes method is 4.8352 g/cm^3^, about 96.74% of the theoretical density. However, as the temperature further increases to 1500 °C and 1525 °C, as shown in Figure 3d,e, it is observed that the size of the grain is abnormally large, about two times larger than that in 1475 °C. This phenomenon may result from the secondary grain growth caused by high sintering temperature, which is unfavorable for the uniformity of grain size distribution.

### 3.3. Bond Traits and Microwave Dielectric Performances Investigation of Sr_2_TiO_4_ Ceramic

**Figure 4:**
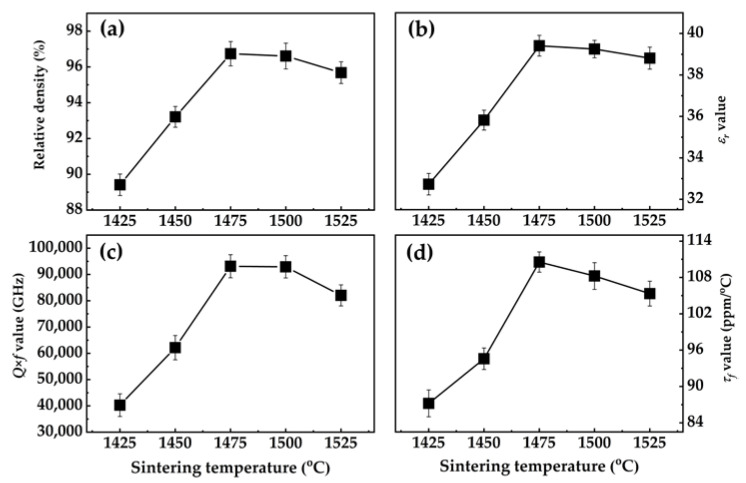
Development of (**a**) relative density, (**b**) *ε_r_* value (measured in the ranges of 4.8~5.2 GHz), (**c**) *Q × f* value, and (**d**) *τ_f_* value.

The Sr_2_TiO_4_ ceramics are sintered at 1425~1525 °C, and the developments of densifications and microwave dielectric performances are shown in Figure 4. Firstly, from Figure 4a, it is found that the Sr_2_TiO_4_ ceramics reach high densifications (>95%) at 1475 °C. The variations of *ε_r_* value are dominated by extrinsic and intrinsic factors, such as densification, dielectric polarizability, and phase compositions [18]. For instance, in this study, the dielectric polarizability remains unchanged since there is no additional ionic dopant. Moreover, the phase structure is the same tetragonal Ruddlesden–Popper type. Thus, the *ε_r_* value in Figure 4b presents a comparable trend with the relative density. Figure 4c shows that the *Q × f* value increases to 93,120 GHz at 1475 °C and declines afterward. It is widely acknowledged that the *Q × f* value is sensitive to grain growth, densification, and phase constitutions [19]. In our present study, since the phase structure remains unchanged, the densification and growth of grain is important for the development of the *Q × f* value. Combined with the SEM images and variation in average grain size, it is clearly observed that the grains are fully grown, and the porosity of the microstructure is reduced, which is beneficial for reducing the number of grain boundaries per unit volume, reducing external grain boundary losses, and then increasing the *Q × f* value. However, excessive sintering temperature causes abnormal grain growth, which disrupts the uniformity of grain size and is not conducive to the improvement of *Q × f* value [20]. The variations in *τ_f_* value in Figure 4d are similar to the evolutionary trends of the *ε_r_* and *Q × f* values, which are also caused by the mutual influences from the densification, phase structure, and dielectric polarizability.

Figure 4 shows that optimum microwave dielectric properties can be acquired when sintered at 1475 °C: *ε_r_* = 39.41, *Q × f* = 93,120 GHz, *τ_f_* = 110.54 ppm/°C. In the optimum state, the impacts of extrinsic loss can be ignored; thus, the intrinsic factor determining the microwave dielectric performances of Sr_2_TiO_4_ ceramics, namely the structure–property connection, should be unambiguously analyzed. The chemical bond theory founded by Philips [21], Van Vechten [22], and Levine [23] (hereafter shortened as P–V–L complex chemical bond theory) deliver a strategy for calculating the fundamental chemical bonds characteristics, such as bond ionicity (*f_i_*), bond covalency (*f_c_*), bond susceptibility (χ), lattice energy (*U*), and the thermal expansion coefficient (*α_L_*). The phase structure characteristics are able to be specifically classified into chemical bond properties within the structure using these bond traits [4,24].

**Figure 5:**
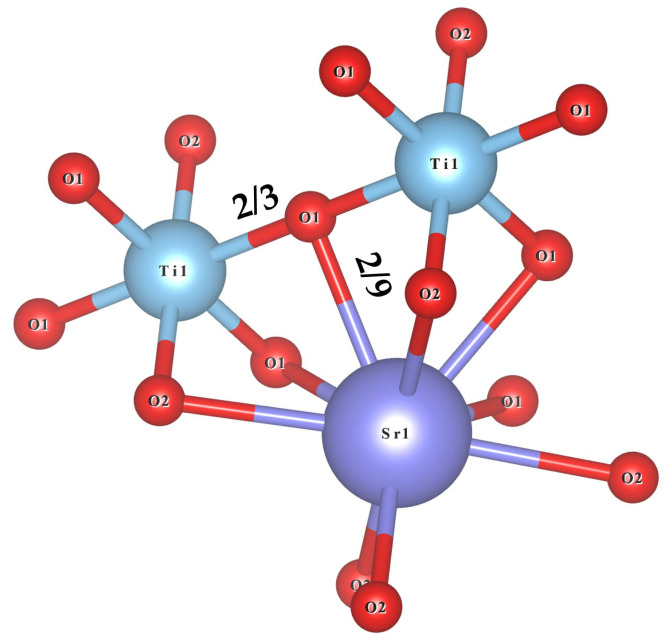
The bonding environment, coordinates, and charge distributions of cations in Sr_2_TiO_4_ ceramic.

Generally, a crystal can be treated as a combination of chemical bonds between ions. The molecular formula of a crystal can also be regarded as the summation of chemical bonds. A chemical bond is binary, and the binary compounds A_m_B_n_ can express its chemical formula; thus, the complex crystal A_a_B_b_C_c_D_d_ is disassembled into the summation of binary crystals based on the crystal structure configuration, molecular formula, and chemical bond types [25]. Followed this, the traits of multi-bonds can be solved by treating them as a single bond. Thus, the binary expressions of Sr_2_TiO_4_ are firstly recognized on the basis of its unique phase structure, coordinating atmosphere, and charge distribution of ions, as shown in Figure 5.

It shall be noted that the effective valence electron numbers (Z) of the O anions in Sr–O and Ti–O bonds are 4 and 12, respectively. Based on the resolution theory of binary crystal formulas for multi-crystalline crystals proposed by Zhang [25], the summation of binary crystals in Sr_2_TiO_4_ is described as follows:

$$\begin{array}{l} Sr_{2}TiO_{4}=Sr_{2}TiO1_{2}O2_{2}\\ Sr_{2}TiO_{4}=Sr_{8/9}O1_{4/3}+Sr_{8/9}O2{\left(1\right)}_{4/3}+Sr_{2/9}O2{\left(2\right)}_{1/3}+Ti_{2/3}O1_{2/3}+Ti_{1/3}O2_{1/3} \end{array}$$

The chemical bonds are not 100% ionic or covalent for an ionic crystal. Thus, it is crucial to distinguish the ionic part and covalent part of chemical bonds in Sr_2_TiO_4_ ceramic. The bond ionicity value of any bond ($f_{i}^{\mu }$) is estimated using the following equations:

(4) $$f_{i}^{\mu }=\frac{{\left(C^{\mu }\right)}^{2}}{{\left(E_{g}^{\mu }\right)}^{2}}$$

(5) $$f_{c}^{\mu }=1-f_{i}^{\mu }$$

(6) $$f_{c}^{\mu }=\frac{{\left(E_{h}^{\mu }\right)}^{2}}{{\left(E_{g}^{\mu }\right)}^{2}}$$

where *C^μ^*, $E_{h}^{\mu }$, and $E_{g}^{\mu }$ are the heteropolar part, homopolar part, and average energy gap of Sr_2_TiO_4_ ceramic, respectively. They are calculated based on the following equations:

(7) $$E_{g}^{\mu }=\sqrt{{\left(E_{h}^{\mu }\right)}^{2}+{\left(C^{\mu }\right)}^{2}}$$

(8) $$E_{h}^{\mu }=\frac{39.74}{r^{2.48}}$$

(9) $$C^{\mu }=14.4\times b^{\mu }\times (Z_{A}^{\mu }-\frac{n}{m}Z_{B}^{\mu })\times \exp\left(-k_{s}r_{0}^{\mu }\right)/r_{0}^{\mu }\;(n>\;m)$$

where $r_{0}^{\mu }$ equals half of the length *d^μ^*, acquired from the Rietveld refinement method; $Z_{A}^{\mu }$ and $Z_{B}^{\mu }$ signify the number of effective valence electrons; $\exp\left(-k_{s}r_{0}^{\mu }\right)$ is the calculated Thomas–Fermi screening index; *b^μ^* is the correction index related with the mean coordination number $N_{c}^{\mu }$, which is obtained as follows:

(10) $$N_{c}^{\mu }=\frac{m}{m+n}N_{cA}^{\mu }+\frac{n}{m+n}N_{cB}^{\mu }$$

(11) $$b^{\mu }=0.089\cdot {\left(N_{c}^{\mu }\right)}^{1.48}$$

where *m* and *n* are gained from the binary bonding formula expression A_m_B_n_, and $N_{cA}^{\mu }$, $N_{cB}^{\mu }$ are the coordination numbers of the *A* and *B* atoms (for Sr, Ti, and O, they are 9, 6, and 6, respectively). The $\exp\left(-k_{s}r_{0}^{\mu }\right)$ is obtained as follows:

(12) $$k_{s}^{\mu }={\left(\frac{4k_{F}^{\mu }}{\pi a_{0}}\right)}^{1/2}$$

where *a*_0_ is the Bohr radius, and $k_{F}^{\mu }$ the Fermi wave vector. The $k_{F}^{\mu }$ is calculated as follows:

(13) $$k_{F}^{\mu }={\left(3\pi^{2}N_{e}^{\mu }\right)}^{1/3}$$

where $N_{e}^{\mu }$ is the effective valence electron density, as obtained from the following:

(14) $$N_{e}^{\mu }=\frac{n_{v}^{\mu }}{v_{b}^{\mu }}=\frac{\left(\frac{Z_{A}^{\mu }}{N_{cA}^{\mu }}+\frac{Z_{B}^{\mu }}{N_{cB}^{\mu }}\right)}{V_{b}^{\mu }}$$

(15) $$V_{b}^{\mu }=\frac{{\left(d^{\mu }\right)}^{3}}{\sum {\left(d^{v}\right)}^{3}N_{b}^{\mu }}$$

where $n_{v}^{\mu }$, $V_{b}^{\mu }$, and $N_{b}^{\mu }$ are the number of valence electrons, bond volume, and bond density of Sr_2_TiO_4_ ceramic, respectively. Therefore, the $f_{i}^{\mu }$ value of chemical bonds is calculated, and the comparisons are shown in Figure 6.

It is found that Sr–O bonds show larger $f_{i}^{\mu }$ values in comparison to Ti–O bonds, demonstrating that Sr–O bonds in [SrO9] polyhedrons may provide a larger contribution to the dielectric polarizations within Sr_2_TiO_4_ ceramic. Nevertheless, the theoretical calculation of dielectric polarizations shall be evaluated to comprehend chemical bond contributions better. The bond susceptibility (*χ^μ^*) in P–V–L complex chemical bond theory reflects the dielectric polarizations of any bond, which is defined as follows [23]:

(16) $$\chi_{}^{\mu }=\frac{{\left(\hbar \Omega_{p}\right)}^{2}}{4\pi {\left(E_{g}^{\mu }\right)}^{2}}$$

(17) $$\chi =\sum F^{\mu }\times \chi^{\mu }$$

where ℏ and Ω*_p_* represent Planck’s constant and plasma frequency of bond, respectively; *F^μ^* is the proportion of *μ* type of bonds in all the bonds. The calculated *χ^μ^* and proportions *χ^μ^*/*χ* are listed in Table 3. It is also found that the largest contributions to the dielectric polarization come from Sr–O2(1) and Sr–O1 bonds. Moreover, Sr–O bonds contribute about 62.13% to the dielectric polarization, greater than the Ti–O bonds (37.87%). This result confirms the conclusion of bond ionicity analysis, showing that the Sr–O bonds may be more important in determining the dielectric polarization.

**Figure 7:**
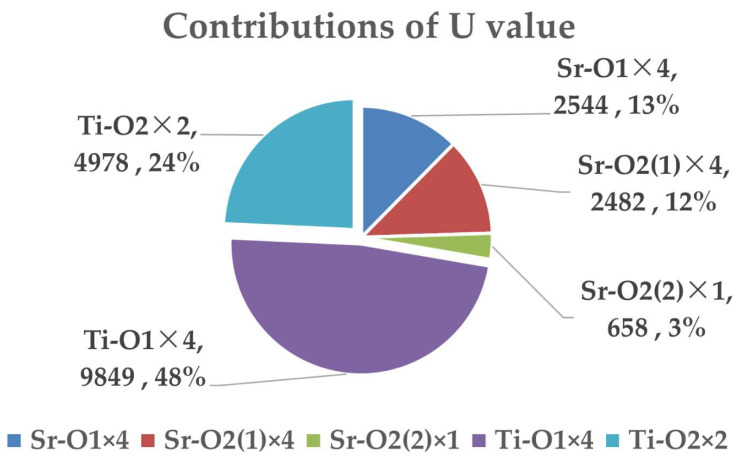
Contributions of chemical bonds to the lattice energy *U* value.

The *Q × f* value is closely connected with the dielectric loss tan*δ* (*Q* = 1/tan*δ*, *f* is the resonant frequency), which is considered from the inherent and extrinsic loss. Extrinsic loss refers to the size of grain growth, densification, and phase constitutions, etc. [26]. Internally, non-harmonicity of lattice vibrations in a faultless crystal produces intrinsic loss [27]. Lattice energy (*U*) indicates the binding abilities between ions [28]. A phase structure shows high stability with a strong binding ability [29]. The *U* value is analyzed as follows:

(18) $$U_{total}=\sum_{\mu }\left(U_{bc}^{\mu }+U_{bi}^{\mu }\right)$$

(19) $$U_{bc}^{\mu }=2100\times m\frac{{\left(Z_{+}^{\mu }\right)}^{1.64}}{{\left(d^{\mu }\right)}^{0.75}}f_{c}^{\mu }$$

(20) $$U_{bi}^{\mu }=1270\frac{\left(m+n\right)Z_{+}^{\mu }Z_{-}^{\mu }}{d^{\mu }}\left(1-\frac{0.4}{d^{\mu }}\right)f_{i}^{\mu }$$

where $Z_{+}^{\mu }$ and $Z_{-}^{\mu }$ are the valence states of the Sr^4+^, Ti^4+^, and O^2−^, *d^μ^* is the distance between cation and anion, and the *m*/*n* value is gained from the binary bonding formula. The calculated *U* value and the contributions of chemical bonds are shown in Figure 7. As we can see, the *U*_Ti–O_ is approximately three times greater than *U*_Sr–O_, which emphasizes that the Ti–O bonds play a dominating role in the lattice stability and Ti–O bonds are more crucial in regulating the intrinsic dielectric loss.

The temperature coefficient of resonance frequency (*τ_f_*) reflects the temperature stability of the ceramic system in variable conditions. It shall be properly adjusted based on practical application. From the previous literature, the *τ_f_* value is affected by the effects of dielectric polarization capacity and the coefficient of thermal expansion as follows [30]:

(21) $$\tau_{f}=-\left(\alpha_{L}+\frac{\tau_{\varepsilon }}{2}\right)$$

where *α_L_* represents the thermal expansion coefficient, and *τ_ε_* stands for the permittivity temperature coefficient. The *τ_f_* value is inversely proportional to the *τ_ε_* and *α_L_* values. The *τ_ε_* is typically affected by dielectric polarizability and increases with the decline in the *ε_r_* value [31]. Based on the P–V–L complex chemical bond theory, the *τ_f_* value is inversely proportional to the *α_L_* value. The *α_L_* is created by the anharmonicity in the Sr_2_TiO_4_ phase structure [32], which is calculated by using the estimated lattice energy *U*, as follows:

(22) $$\alpha =\sum F_{mn}\times \alpha_{mn}^{\mu }$$

(23) $$\alpha_{mn}^{\mu }=-3.1685+0.8376\gamma_{mn}$$

(24) $$\gamma_{mn}=\frac{kZ_{A}N_{CA}{}^{\mu }}{U\left(A_{m}B_{n}\right)\Delta A}\beta_{mn}$$

(25) $$\beta_{mn}=\frac{m\left(m+n\right)}{2n}$$

where *F_mn_* is the proportion of this type of chemical bond in the Sr_2_TiO_4_ phase structure. *k* and Δ*A* are the Boltzmann constant and correction index, respectively. The results are presented in Table 4. It is found that the *α_L_* value of the Sr_2_TiO_4_ structure is estimated at about ~16.45 ppm/°C, and the Ti–O bonds are more significant than Sr–O bonds.

## 4. Conclusions

This study mainly investigates the crystal structure, bond characteristics, and structure–property relationship of Sr_2_TiO_4_ ceramics. The Sr_2_TiO_4_ ceramic sintered at 1475 °C shows a compact microstructure and great microwave dielectric performances, namely an *ε_r_* value of 39.41, a *Q × f* value of 93,120 GHz, and a *τ_f_* value ca. of 110.54 ppm/°C. Under the optimum sintering temperature, the extrinsic factors can be excluded, and the structure’s configuration mainly decides the structure–property relationship. On the basis of the P–V–L complex chemical bond theory, it is shown that the Sr–O bonds have larger average bond ionicity (*f_i_*) values (about 62.2538%) than Ti–O bonds (about 56.6779%). Also, the bond susceptibility (*χ^μ^*) value of Sr–O bonds indicates their contribution of about 62.13% to the dielectric polarization, which is also greater than the Ti–O bonds (37.87%). This result demonstrates that the Sr–O bonds are the main factors contributing to the dielectric polarizability. The largest lattice energy (*U*) value of Ti–O bonds, however, about three times larger than Sr–O bonds, clarifies their significance in structural stability. The Ti–O bonds are also crucial for developing the thermal expansion coefficient value of the Sr_2_TiO_4_ structure, which is important for the development of the temperature coefficient of resonance frequency.

## Figures and Tables

**Figure 6:**
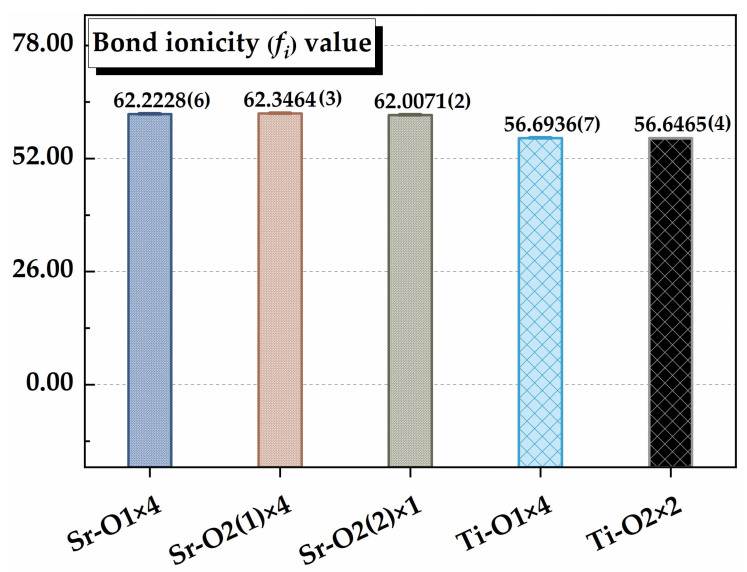
The bond ionicity $f_{i}^{\mu }$ value of chemical bonds in the Sr_2_TiO_4_ structure.

**Table 1:** Reported microwave dielectric material candidates with medium *ε_r_* (20~70) values [5,6,7,8,9,10,11].

System	ST (°C)	*ε_r_*	*Q × f* (GHz)	*τ_f_* (ppm/°C)	Ref.
Nd_1.02_(Nb_0.94_Ta_0.06_)_0.988_O_4_	1275	21.7	51,000	−45.7	[5]
Zn_1.01_Nb_2_O_6_/TiO_2_/Zn_1.01_Nb_2_O_6_	1200	26.8	99,500	0.5	[6]
ZnTi_0.7_Ge_0.3_Nb_2_O_8_	1120	35.6	62,700	−58.0	[7]
0.516ZTN–0.484ZNT	1100	46.1	27,031	−1.5	[8]
Co(Ti_0.8_Zr_0.2_)Nb_2_O_8_	1250	55.0	41,541	59.8	[9]
Sr_3_Ti_2_O_7_	1500	63.0	84,000	293.0	[10]
Cu_0.5_Ti_0.5_NbO_4_	960	71.2	11,000	49.2	[11]

**Table 2:** Atomic fractional coordinates of Sr_2_TiO_4_ ceramic system.

Atom	Position	Fractional Coordinates			Occ ^1^	U_iso_ ^2^
		*x*	*y*	*z*		
Sr	4c	0.000	0.000	0.355 (4)	1	0.00988 (5)
Ti	8d	0.000	0.000	0.000	1	0.00387 (6)
O1	8d	0.000	0.500	0.000	1	0.02071 (8)
O2	8d	0.000	0.000	0.152 (3)	1	0.02512 (4)
Bond lengths		Sr–O1 × 4: 2.66932 (2) Å Ti–O1 × 4: 1.94490 (10) Å Sr–O2(1) × 4: 2.75192 (4) Å Ti–O2 × 2: 1.91655 (3) Å Sr–O2(2) × 1: 2.55960 (10) Å				

^1^ Occ: site occupancy; ^2^ U_iso_: isotropic atomic displacement parameters.

**Table 3:** Chemical bond susceptibility in the Sr_2_TiO_4_ structure.

Bond Type	$N_{e}^{\mu }$	$E_{g}^{\mu }\;(\mathrm{eV})$	*Z_O_^μ^*	*χ^μ^*	*F^μ^*	*χ^μ^*/*χ* (%)
Sr–O1 × 4	0.193981 (7)	5.66421 (4)	4	7.415 (8)	0.333	27.12 (3)
Sr–O2(1) × 4	0.177033 (5)	5.26030 (6)	4	7.859 (3)	0.333	28.74 (8)
Sr–O2(2) × 1	0.220011 (8)	6.26772 (3)	4	6.854 (1)	0.083	6.27 (2)
Ti–O1 × 4	1.504495 (3)	11.6008 (2)	12	13.933 (5)	0.167	25.48 (2)
Ti–O2 × 2	1.572252 (4)	12.0245 (5)	12	13.547 (7)	0.083	12.39 (3)

**Table 4:** Thermal expansion coefficient in the Sr_2_TiO_4_ structure.

Bond Type	$F_{mn}^{\mu }$	*U* (kJ/mol)	$\alpha_{mn}^{\mu }\left(10^{-6}\cdot K^{-1}\right)$	α (10^−6^ K^−1^)
Sr–O1 × 4	0.333	2544 (8)	20.23 (7)	6.74 (1)
Sr–O2(1) × 4	0.333	2482 (3)	20.81 (2)	6.94 (3)
Sr–O2(2) × 1	0.083	658 (6)	19.46 (5)	1.62 (2)
Ti–O1 × 4	0.167	9849 (5)	4.63 (4)	0.77 (4)
Ti–O2 × 2	0.083	4978 (4)	4.55 (1)	0.38 (1)
α_total_ (10^−6^ K^−1^)				16.45 (11)

## Data Availability

Not applicable.
